# New Discoveries in Radiation Science

**DOI:** 10.3390/cancers13051034

**Published:** 2021-03-02

**Authors:** Géza Sáfrány, Katalin Lumniczky, Lorenzo Manti

**Affiliations:** 1 Department Radiobiology and Radiohygiene, National Public Health Center, 1221 Budapest, Hungary; lumniczky.katalin@nnk.gov.hu; 2 Department of Physics, University of Naples Federico II, 80126 Naples, Italy; manti@na.infn.it

This series of 16 articles (8 original articles and 8 reviews) was written by internationally recognized scientists attending the 44th Congress of the European Radiation Research Society (Pécs, Hungary). Ionizing radiation is an interesting agent because it is used to cure cancers and can also induce cancer. The effects of ionizing radiation at the organism level depend on the response of the cells. When radiation hits a cell, it might damage any cellular organelles and macromolecules. Unrepairable damage leads to cell death, while misrepaired alterations leave mutations in surviving cells. If the repair is errorless, normal cells will survive. However, in a small percentage of the seemingly healthy cells the number of spontaneous mutations will increase, which is a sign of radiation-induced genomic instability. Radiation-induced cell death is behind the development of acute radiation syndromes and the killing of tumorous and normal cells during radiation therapy. Radiation-induced mutations in surviving cells might lead to the induction of tumors.

According to the central paradigm of radiation biology, the genetic material, that is the DNA, is the main cellular target of ionizing radiation. Many different types of damage are induced by radiation in the DNA, but the most deleterious effects arise from double strand breaks (DSBs). Unrepaired DSBs lead to cell death during mitosis, therefore it is essential for cellular survival that DNA integrity is restored before the next mitosis. Radiation-induced DSBs are rapidly recognized in the cells by the ataxia telangiectasia mutated (ATM) protein. ATM exhibits serine/threonine kinase activity and through phosphorylation of various proteins, it initiates a cascade of events for the processing of DSBs. It is suggested that in higher eukaryotes, there are four different pathways repairing DSBs: [1]. (1). Homologous recombination (HR) repair corrects DSBs in an error-free manner. (2). Despite of the error-free operation of HR, the most frequently applied mechanism for the correction of DSBs is non-homologous end joining (NHEJ) repair. NHEJ is very fast, but it usually leaves errors in DNA such as additions or deletions of nucleotides, translocations and chromosome rearrangements. (3). Alternative end-joining is slower and even more error-prone. (4). Single-strand annealing is associated with large deletions and may also form translocations. It is suggested that the degree of chromatin destabilization will determine the pathway applied for the repair of DSBs [1].

There are certain chromosomal aberrations, such as dicentric and ring chromosomes, arising because of the non-correctly repaired radiation-induced DSBs. The formation of these chromosomal aberrations can be used to study the efficiency of double strand break repair and for bio-dosimetry purposes to estimate absorbed radiation doses. Ricoul et al. [2] combined the Premature Chromosome Condensation (PCC) technique with γ-H2AX immunofluorescence staining to follow the kinetics of DNA damage and chromosome repair. γ-H2AX, an ATM phosphorylated H2AX histone, binds to DSBs and can be used to locate double-strand breaks and follow the repair processes within cells. According to Ricoul et al., chromosome repair occurs in two steps. There is an early step of DNA DSB joining within three hours after irradiation, when aberrant chromosomes are formed, that is followed by a second, slower, error-free repair mechanism that occurs for 24 h, which restores chromosome integrity [2].

Deficiencies in repair factors of HR or NHEJ might lead to chromosomal catastrophic events and stimulate chromothripsis characterized by genomic rearrangements. Pantelias et al. [3] proposed that PCC dynamics might participate in the induction of chromothripsis. To test this hypothesis, they irradiated cells to create micronucleated cells and applied the PCC technique to get asynchronous multinucleated cells. Using a selective ATP-competitive inhibitor of CDK1, cell cycle arrest was initiated at the G2/M boundary to delay cells entering mitosis. In this way, they were able to demonstrate that asynchrony between micronuclei and main nuclei was an important determinant of chromosome shattering and thus chromothripsis [3].

One of the intriguing open questions in radiation science is the problem of individual radiation sensitivity. In a review paper, Berthel et al. [4] discussed the role of the ATM protein in individual radiation responses. They propose that ATM is present in a dimeric autophosphorylated form (pATM) in the cytoplasm and irradiation induces the monomerization of ATM dimers in a dose-dependent manner. Then, ATM monomers can diffuse into the nucleus and phosphorylate H2AX histones to initiate DSB repair, namely NHEJ. After the completion of repair, pATM is formed again by dimerization. Any delay in the Radiation Induced ATM NucleoShuttling (RIANS) leads to radiosensitivity and genomic instability. A study performed by the authors demonstrated that the number of nuclear pATM foci efficiently predicts radiosensitivity. The authors use the RIANS model to explain the potential cellular backgrounds of the linear-quadratic model, low dose hypersensitivity and adaptive responses [4].

Beside human studies, experimental model systems might also supply important information on the molecular pathways influencing radiation sensitivity. Tardigrades, for instance, are small aquatic animals which are extremely resistant to desiccation and to various agents including ionizing radiation. The LD50 values of x- and γ-rays are three orders of magnitude higher in tardigrades (3–5 kGy) than in humans (3.5–4 Gy). Current research on radiation response of tardigrades suggests that mechanisms protecting DNA damages and initiating DNA repair pathways both contribute to the extreme radiation resistance. Cancer research might also benefit from tardigrades studies investigating molecular pathways involved in genome integrity [5].

It is well known that radiation-induced toxic, early- and late normal tissue effects will develop in about 5–10% of cancer patients undergoing radiation therapy. Normal tissue toxicities severely limit the doses which might be delivered to tumors; therefore, therapeutic regimens are adjusted not to tumor cures, but to an acceptable level of normal tissue sequels. A review paper of De Leve et al. summarizes normal tissue toxicities in different organs both in humans and preclinical models [6]. Special consideration is paid to the development of biological approaches which may specifically limit normal tissue toxicities or increase radiation sensitivities of tumor cells. One biological approach might focus on extracellular adenosine, which is a critical endogenous mediator for the maintenance of homeostasis in various tissues. The immunoregulatory CD73/adenosine system might have an important role in radiation-induced fibrotic disease in normal tissues. De Leve et al. suggests that radiation-induced activation of CD73/adenosine signaling might promote radiation-induced normal tissue toxicity and indicate that the activity of the CD73/adenosine system in the tumor environment may increase tumor growth and tumor immune escape as well. The authors discuss the potential inhibition of CD73/adenosine-signaling pathway as a promising strategy to improve the therapeutic effects of radiation therapy [6].

In the context of radiation-induced systemic immune system effects, immune and inflammation-related parameters were studied in the peripheral blood of head-and-neck cancer patients undergoing radiation therapy before and at two different time points after therapy. It was reported that a certain degree of immune suppression was induced by the malignant condition exhibiting increased number of regulatory T cells and increased levels of CTLA4 and PD-1 expressions on CD4 cells. These alterations were augmented by radiation therapy. Expression of FXDR, SESN1, GADD45, DDB2 and MDM2 radiation-response genes were altered in the blood cells of patients after therapy. These changes were detectable even 1 month after therapy. It is suggested that these markers might help the stratification of head and neck cancer patients [7].

If one could predict the development of toxic sequels before the onset of radiation therapy, then radiation regimens could be adjusted to individual needs. Unfortunately, none of the currently available predictive assays are suitable to estimate individual responses to radiation. In an attempt to establish a reliable predictive marker, Medipally et al. [8] studied the potential of Fourier transform infrared (FTIR) spectroscopy to follow radiotherapy responses. They collected blood samples from prostate cancer patients before, after and at various stages of radiation therapy and recorded the FTIR spectra. The authors observed differences in patients presenting minimal and severe early and late sequel. It is suggested that the application of this technology might provide information for the individualization of radiation therapy [8].

Beside clinical trials, animal studies also present essential information on the development of normal tissue reactions; however, the application of different scoring systems for the evaluation of side effects in patients and preclinical models might severely affect the reliable comparison of the studies. In an attempt to develop a comparative scoring system, Dombrowsky et al. [9] irradiated the ear pinnae of mice with different hypofractionated doses and followed acute (ear thickness, erythema, desquamation) and late (chronic inflammation, fibrosis) effects, as well as the presence of transforming growth factor beta 1 (TGF1)-expressing cells in the radiation field. The authors concluded that ear thickness can be used to describe the severity of early reactions and to predict late sequels [9] in rodent models.

Because of the relatively high costs of murine models, Dünker et al. [10] discussed the potential application of a chicken chorioallantoic membrane (CAM) model in preclinical studies of the therapeutic effects of ionizing radiation. In a review paper, the authors summarize current knowledge on the CAM assay, and analyze its biological, technical and ethical advantages. According to the authors, the CAM assay allows high flexibility in experimental design and can be used to compare the efficiency of different radiation schedules and qualities, to investigate normal and tumor tissue responses, to evaluate the effects of hypoxia and to analyze the potential involvement of the immune system in radiation reactions [10].

Radiation-induced toxic late effects develop because radiation damages the stem cells or the capillary endothelial cells of the affected tissues. Konířová et al. [11] investigated radiation effects on neuronal stem cells (NSCs) isolated from ventricular-subventricular zone of mouse brain. They report here that the number of γ-H2AX foci representing radiation-induced DSBs increased in a dose dependent manner in in vitro cultured neuronal stem cells and the expression of genes involved in DNA damage response (CDKN1A, GADD45A) also increased. Irradiation of NSCs resulted in a mild increase of apoptosis, while radiation promoted NSC differentiation [11].

Radiotherapy is arguably effective at locally controlling cancer for many patients, thereby representing a formidable tool for cancer cure. The metastatic ability of cancer cells, however, defined as the migration of cancer cells from the primary site, is one of the main causes for treatment failure, accounting for almost 90% of cancer patient deaths. It is a complex process, assisted by metabolic changes in the tumor microenvironment and influenced by hypoxia and angiogenesis, whereby cancer cells may acquire a motile phenotype promoting their invasiveness. Radiation is known to modify the tumor microenvironment, which is a complex milieu where cancer cells coexist with normal and stem cells. Kadhim et al. [12] reviewed in vitro and in vivo evidence for the possible influence that radiation may have on the metastatic potential of breast cancer cells through both direct and indirect effects. The former refer to those in directly damaged but surviving cancer cells, while the latter reflect the myriad of factors released by such cells and modifying the behavior of neighboring cells, such as exosomes, nanovesicles that are key mediators in cell-to-cell communication. Such effects include the promotion of epithelial-mesenchymal transition and changes in glycosylation of cell surface proteins. The authors highlight how the benefits of breast cancer radiotherapy is well ascertained and hence must not be overshadowed by the still unclear role that treatment may have in enhancing the metastasis process, for which existing evidence must be carefully evaluated and further data acquired.

New radiotherapy beam delivery techniques are continuously tested in experimental set ups and in clinical trials. Some of the new techniques, for instance laser-accelerated electrons, apply pulsed radiation where the evaluation of the experimental outcomes is challenged by fluctuating beam intensities resulting in deviations from the prescribed dose. In these cases, one should use statistical methods for the analysis of the outcomes, which allow the inclusion of data points with deviations in dose delivery. Using experimental and simulated tumor-growth data, Karsch et al. [13] compared the biological efficacy of laser-driven and conventional clinical Linac electrons with four statistical analysis methods including the classical averaging per dose point, multivariable linear regression, Cox regression and a Monte-Carlo-based approach. The first method excludes animals with high dose deviations, while the other methods include all animals in the analysis. Although all of the approaches resulted in a comparable radiobiological efficiency, the Monte-Carlo-based method, the linear regression and Cox regression were more sensitive than the conventional method and should be used in future studies [13].

As mentioned above, the peculiarity of ionizing radiation lies in its dual nature: being a potent carcinogen and an effective cancer treatment modality. The most extensive and robust evidence supporting the carcinogenic ability of radiation derives from the well-known Life-Span (LSS) study on the atomic bomb survivors of Hiroshima and Nagasaki. Further data continue to be generated from retrospective epidemiological studies on cancer incidence in cohorts of individuals involved in accidental exposures such as the Chernobyl nuclear accident and may contribute to shed light on cancer risk in the low-dose region where LSS-based risk projections have been extrapolated. In this context, Bazyka et al. [14] published new data from the monitoring of Chernobyl cleanup workers, evacuees and other members of the general public in Ukraine over the 1990–2016 periods. The authors report that a significantly elevated thyroid cancer incidence was identified in the male cleanup workers’ cohort (150,813 individuals) for the 1986–2012 period, with an overall standardized incidence ratio (SIR) of 3.35 (95% CI: 2.91–3.80). To connect such specific subtypes of radiogenic cancers with radiation exposure, a set of molecular investigations was also performed among the cohort members, looking at gene expression, telomere length, γ-H2AX and Cyclin D1. Alterations in gene profile after exposure could represent background biomarkers for late effects from low-dose exposure. The authors found a statistically significant and dose-dependent decrease in expression of the BCL2, SERPINB9, CDKN2A and STAT3 genes in parallel to a dose-dependent overexpression of MCF2L and upregulation of TP53 (up to 100 mSv). Hyper expression of TNF gene in doses above 100 mSv to 1000 mSv was also found. Finally, an increased expression of γ-H2AX and Cyclin D1 correlated to radiation dose, telomere shortening to age, and concomitant pathology. In conclusion, 30 years after the Chernobyl accident in cleanup workers, an excess was demonstrated in incidences of the “early” cancers—thyroid, breast and leukemia—with a dose dependency for thyroid cancer and leukemia; for breast cancer incidence, there were indications of an increase, though the data could not be considered conclusive. In the general population of the Zhytomyr and Kyiv regions of Ukraine and in a cohort of evacuees from Pripyat and Chernobyl towns, only the incidence of thyroid cancers increased.

Although the central dogma of radiation action at the cellular level is based on the notion that, for biologically relevant effects to arise, the DNA of irradiated cells ought to be damaged, it is established that non-targeted effects (NTEs) may play an import role. NTEs are defined as those manifesting themselves in cells that did not receive direct energy deposition but as a result of factors released by directly exposed cells affecting nearby cells (bystander effects) or of the destabilization of the DNA that surfaces in the progeny of irradiated cell populations as de novo damage, quantitatively not reconcilable with that induced initially and inherited by proliferating surviving cells (genomic instability). Controversy still exists on the in vivo relevance of radiation-induced NTEs, especially in the radiotherapy scenario. It is highlighted that the magnitude and quality of NTEs differ between tumor and noncancerous cell/cell lines, with prevalence in normal cells [15]. This has prompted Mothersill et al. to suggest that decreasing NTEs in normal cells may result in an improvement of the radiotherapy therapeutic window. In addition, the authors explored the NTE relevance in the diagnostic scenario: in fact, it is well-established that NTEs are particularly manifest in the low dose region, with in vitro studies demonstrating the acute onset of bystander and other NTEs for doses as low as a few mGy [15].

Another category of effects ascribed to NTEs are the so-called transgenerational effects of parental exposure to mutagens such as radiation. The study of defects at birth in the progeny of individuals exposed to radiation has been traditionally used as a tool to estimate the risk of inheritable effects or radiation due to genome-wide mutations. However, as Dubrova [16] pointed out, results from both the Hiroshima and Nagasaki survivors’ databases as well as those from patients subjected to radiotherapy failed to provide evidence for such effects. This is, however, at odds with data obtained from irradiating germline cells in rodents, where such effects were highlighted by whole-genome sequencing and attributed to epigenetic mechanisms. Therefore, more sensitive and sophisticated investigative tools recently made available by developments in whole-genome sequencing are invoked to improve our knowledge on the hereditary effects of radiation effects in humans.
